# Specific Causes of Oxyphosphate Cement Disintegration

**Published:** 1908-06-15

**Authors:** J. O. Keller

**Affiliations:** Chicago, Ills.


### SPECIFIC CAUSES OF OXYPHOSPHATE CEMENT DISINTEGRATION.

By J. O. Keller, D. D. S., Chicago, Ills.

A reasoning understanding of the physics and chemistry of the powders and liquids and resultants cement is necessary to a solution of this problem.

Zinc oxide (molecules, one atom of zinc and one atom of oxygen,) is the main, and most generally the entire, constituent of dental cement powders. By calcination to about 2,000 degrees, these molecules are concentrated into compound molecular, small, soft, flour-like, flaky particles. These soft flaky particles, when fused together at high heat, about 2,800 degrees, become hard, vitrified, grit, glass-like mass.

Laboratory experiments show that low fusion powder particles of zinc oxide, either in powder or cement mass, are readily acted upon by such acid and acrid fluids as are found in the salivary secretions, and that in proportion as hardness, concentration and vitrifaction by high heat is gained, they resist the action of such acids and acrid agencies; also that the soda of the soda phosphate combines readily with them.

### PHYSICAL AND CHEMICAL TECHNIC.

The forces which hold together the structure of cement masses are physical and chemical, one physical and two independent chemical forces.

The cement mass is made up of the following units, not including its elementary composition, as follows:

First, zinc oxide molecules as bought in a crude shape in the drug shops, and afterward calcined into low fusion or high fusion zinc oxide masses. The low fusion will be in flour-like, flaky condition, and the high fusion in flour grit-like particles, or coarser, according as pulverized. Second, phosphoric acid. Third, water holding phosphoric acid in solution. Fourth, aluminum phosphate, soda phosphate, zinc phosphate, or other phosphates, either alone or combined severally as may be required. At least one phosphate must be used. Several may be used.

These bonds of union and forces in the cement mass are as follows:

First—The atoms in the zinc oxide molecules, one each of zinc and oxygen, are held together by the force of chemical adhesion.

Second—The force which holds together the molecules of zinc oxide in the calcined particles is called the force of physical cohesion. This force holds together units of the same character.

Third—The chemical units in phosphoric acid and its water diluent and its phosphates are entirely chemical, hence the force which holds them together may be called chemical adhesion, chemism or chemical affinity.

Fourth—Water is a chemical, each molecule containing two atoms of hydrogen and one of oxygen. It is a high phosphoric and phosphate solvent, and the agency of crystallization in dental cement masses.

Phosphoric acid and water are the basic constituents of all oxyphosphate cement liquids—Soda, aluminum, lime, magnesia, and other ingredients may be incorporated by chemical reaction, and become phosphates accordingly. Soda is most always used to neutralize and regulate the setting, without which or other neutralizer, the powder and liquid, when mixed together, would set violently and with high heat. An aqueous solution of glacial phosphoric acid (rightly named glacial soda phosphate) is the usual and easiest way to make a cement liquid. It will contain from 50 to 70 per cent of impurities in the form of soda phosphate, from 5 to 20 per cent of free phosphoric acid, balance water, free and combined. In chemical union. with calcined zinc oxide cement powder, the resultant cement would be double phosphate of soda and zinc.

### SOLVENT CONDITIONS OF THE INTEGERS OR UNITS OF STRENGTH IN OXYPHOSPHATE CEMENT MASSES.

Zinc oxide is practically insoluble in water, requiring 700 parts to hold it in solution. Various acids in water solution will attack zinc oxide readily and chemically combine with it.

Magnesia is very insoluble in water, requiring 10,000 parts to hold it in solution. Soda is very soluble in water, requiring but one part by weight to hold it in solution. Soda phosphate is so very soluble in water that one pound of it will be held in solution with eight ounces of water. Phosphoric acid is so very deliquescent that seven pounds of it will be dissolved in one pound or one pint of water. The metal aluminum is absolutely insoluble in water. Its oxide will not dissolve in 5,000 parts of water. Aluminum phosphate will not completely dissolve in 100 parts of water, the exact percentage not having been determined.

These solvent conditions should be kept in mind, because the degree of solubility in water, in a measure, determines the degree of solubility or attack by the acid or alkaline reagents in the mouth. Water is the means of conveying the diluent acid and alkaline substances not only into contact, but also into the porous cement structure.

Zinc oxide, soda, phosphoric acid and water, are the main constituents of dental cements; hence cement masses consist of molecules or particles of zinc oxide held together in chemical union by a phosphatic liqiud, formulated as aforementioned.

An oxyphosphate cement mass holding crowns and bridges used as filling therefore are double, triple or quadruple phosphates or higher in series according to the number of phosphates incorporated. With aluminum as a phosphate in the liquids, and a zinc powder, the result would be a double phosphate of zinc and aluminum in a cement mass. A combination of magnesia, soda and aluminum phosphates in the liquid and zinc oxide powder would make a quadruple phosphate, magnesia, soda, aluminum and zinc in the cement mass.

### CRYSTALLIZATION.

Cement masses, made with low fusion powders, begin to crystallize as soon as the powder and liquid are mixed. It will expand during the first twenty-four hours, contract the next twenty-four hours and during the next twenty-four or forty-eight hours will expand more than the contraction and expansion of the preceding forty-eight hours. These movements result from a crystallizing process. It may continue for several months, after the cement has apparently set. The crystal formation is sometimes so extensive that the whole cement mass becomes porous, and with open spaces throughout its entire structure. It may be more properly called a crystal porosity. The whole mass of low fusion powder cements undergo complete or body crystallization. High fusion cement masses, that is, cement masses made with high heat calcined powders, undergo but partial crystallization because of the size of the particles. They will have consequent less bulk changes, and crystal porous structure.

Oxyphosphate cement masses are not liable to physical solubility or solubility in water. Cement pellets made with high and low fusion powders did not dissolve during four years in water at ordinary room temperatures, nor during twenty-four hours in boiling water.

### CHEMICAL DISINTEGRATION.

Cement masses of zinc and other phosphates are soluble only in the acid, alkaline and acrid chemical agencies of the mouth, as herein shown.

### PHYSICAL AND CHEMICAL EXPERIMENTS.

High fusion powders require less than half as much liquid as low fusion powders—40 grains of low fusion powder were mixed with enough liquid to make a thick flow, suitable for crowns and bridges. The resultant cement mass weighed 85 grains. Forty grains of high fusion powder required only 20 grains to make a similar flow and consistency. Considering the soluble tendency of the phosphates, this experiment shows at least one reason why cements made with low fusion, flaky, flour-like powders will be more soluble in the salivary secretions, than cement masses made with high fusion powder.

A thin flow mix, suitable for inlay work, required 30 grains of liquid for high fusion and 60 grains for low fusion powders, 40 grains of powder being used each mix. Double the quantity is required for the low fusion powders. A thick mix suitable for filling work required 10 grains of liquid to 40 grains of high and 15 grains of liquid to 40 grains of low fusion powder. Hence the importance of using high fusion powders to gain strength, less solubility, and greater stability of cement structures.

### PHYSICAL AND CHEMICAL CONSTITUTION OF THE SALIVA.

The salivary secretions contain about 986 parts of water to the thousand general average. They are impregnated with various salts, acids, alkalies, and organic matters, which make the oral cavity one of the most intricate, physical and chemical laboratories in the whole economy. The saliva may be impregnated with lactic, acetic, muriatic, sulphuric, uric, oxalic, malic, and other acids, either free or combined, and salts may be found either in the healthy state or indicative of some diseased action. The acidity is sometimes so excessive as to corrode the gums and lips, to set the teeth on edge, and make them sensibly rough to touch and appearance. It may be excessively acid one day and extremely alkaline the next.

Saliva contains frequently, either free or combined, such alkaline substances and salts as potassium chloride, lactates, muriates and phosphates of potash, lime and soda. Sulphocyanide of potassium is frequently abundant. It is sometimes highly charged with sulphurated hydrogen. This gas is harmless of itself, but when absorbed by the salivary secretions of the mouth causes them to become very acid or acrid. This acridity is frequently so strong as to corrode and blacken amalgam fillings, and to tarnish (not corrode) gold fillings in a few days.

The ultimate crystal porosity of many cements causes them to absorb the salivary secretions into their crystal spaces. The water in the saliva carries with it the various acids, alkalies and salts with which it is impregnated, not only in contact with the cement mass, but into its porous crystal structure. Water is not only the agent of crystallization, but carries the agency of solution or disintegration into the mass. By direct chemical action or by chemical reaction, or by acrid erosion, oxyphosphate of zinc cements are dissolved in the mouth by both acid and chemical reactions, as follows:

### ACID CHEMICAL REACTION.

If any acids in the oral fluids have a greater affinity for the soda and zinc oxide (or other phosphate base) in the soda, zinc phosphate cement mass than the phosphoric acid, with which they are in chemical union, then such acids will attack, eat, dissolve or combine with said soda and zinc, enter into chemical union with them, liberate the phosphoric acid, and the filling gradually dissolves. Malic, uric, oxalic, lactic and some other acids are always found in the salivary secretions, either free, or combined. It has been shown by laboratory tests that these acids will dissolve dental cements structure.

### ALKALINE CHEMICAL REACTION.

If any alkaline substance in the saliva has a greater affinity for the phosphoric acid than said acid has for the zinc, soda, aluminum, magnesia, or other oxide base with which it may be combined, then said alkaline agent will chemically unite with the phosphoric acid, liberate the zinc and other oxides, and the cement gradually dissolves. Potash, sulphocyanide of potassium, potassium chloride, chloride of lime and other alkalies and bases either free or combined, are frequently, some always, present in the salivary secretions. Aqueous solutions of most of these alkalies and bases will readily dissolve cement structure because of their higher affinities for its phosphoric acid, than said acid has for the zinc, soda, magnesia and other bases with which it may be combined in the cement structure. Sulphocyanide has a double reaction. It is somewhat acid in its nature, at least very acrid and is also alkaline. The higher affinity of the phosphoric acid in the dental cement mass for the cyanide in the saliva causes it to surrender its chemical union in the cement mass, which gradually dissolves. Sulphocyanide, being both acrid and basic, renders it liable to act both upon the acid and basic structure in the cement mass. The cyanides will unite with the phosphoric acids and sulphocyanide will eat the soda, magnesia and zinc oxide in the cement structure. This is what is called DOUBLE CHEMICAL REACTION. The lactic and muriatic acids in the lactates and muriates of soda, potash, and magnesia in the saliva have feebler affinities for these bases with which they are in chemical union than they have for the zinc oxide and soda oxide in the cement mass; hence they will give up their chemical union in the salivary secretions, attack and chemically unite with the zinc and soda oxide in the cement mass, liberate the phosphoric acid and the filling gradually dissolves. This double chemical reaction will take place under all circumstances, unless the zinc oxide particles in the cement mass are calcined so hard and strong and tough as to resist the acid agencies of the mouth. Glass-like, fully vitrified, very hard and properly calcined zinc oxide particles will resist disintegration by the acid agencies of the mouth, but the alkaline agencies are still free to combine with the phosphoric acid in the cement structure. Unless the phosphate liquid is so formulated as to reduce the degree of solubility in water and consequent less solubility by its alkaline affinities and thus to render the cement mass less liable to disintegration, cements will continue to dissolve in the salivary secretions.

### THE RELATIVE SOLUBILITY OF CEMENTS.

Under the same conditions, cements made with low fusion powders will dissolve quicker than high fusion powder cements. Acid agencies of the mouth will dissolve cements made with low fusion powders very readily, because the particles are smaller, not condensed by calcination, and in consequence the cement mass is more porous and crystalline in structure. It is more readily acted upon by the acids of the mouth, hence such cement masses wash out quickly. The alkaline agencies of the mouth have a strong affinity for the soda phosphate, which is almost universally used in cements, hence the low fusion powder cement masses are in consequence the more readily dissolved. The high fusion powder cement masses, because of the hard, diamond-like condensed state of its grit particles, will resist the action of the acids in the saliva; hence such cement masses are more durable.—February 1908. *Dental Review.*

